# 基于碳点的色谱分离材料研究进展

**DOI:** 10.3724/SP.J.1123.2023.08013

**Published:** 2023-10-08

**Authors:** Jia CHEN, Hongdeng QIU

**Affiliations:** 中国科学院兰州化学物理研究所, 中国科学院西北特色植物资源化学重点实验室, 甘肃 兰州 730000; CAS Key Laboratory of Northwestern Characteristic Plant Resources Chemistry, Lanzhou Institute of Chemical Physics, Chinese Academy of Sciences, Lanzhou 730000, China

**Keywords:** 碳点, 固定相, 液相色谱, 气相色谱, 毛细管电色谱, carbon dots (CDs), stationary phase, liquid chromatography (LC), gas chromatography (GC), capillary electrochromatography (CEC)

## Abstract

色谱分离的核心是色谱柱,色谱柱的灵魂是色谱分离材料,色谱分离材料的种类和性质直接决定色谱的分离模式和分离性能。碳点作为一类新型的零维碳纳米材料,自2004年被首次报道以来,凭借其广泛的原料来源、低毒性、易于功能化、优异的生物相容性和抗光漂白性等独特性能,已广泛应用于生物成像、发光二极管、传感、催化等领域,并呈现出蓬勃的生机。同时,碳点还具有设计性强、粒径大小适中等优势,将其引入色谱分离新材料中开发高选择性的色谱固定相具有重要意义。本文首先简要回顾了碳点的分类、合成策略、发展历程,然后聚焦于色谱分离材料领域,系统综述了近年来碳点在液相色谱固定相(含亲水色谱、反相色谱、混合色谱、手性色谱等)、气相色谱固定相和毛细管电色谱固定相方面的最新研究进展,特别强调了各类固定相的制备方法及其应用,并对碳点在色谱分离材料领域的发展前景和未来努力方向进行了分析和展望,期望为基于碳点的色谱分离新材料的理性设计及应用提供一些参考。

## Abstract (en)

Chromatographic column is the core of chromatographic separation, and chromatographic separation material is considered the soul of the chromatographic column. The type and characteristics of the chromatographic separation material directly determine the separation mode and performance of chromatographic columns. The development and preparation of separation materials with novel structures and good separation performance is an ongoing hotspot in chromatography research. Given rapid developments in nanoscience and technology, nanomaterials with unique surface functional groups and large specific surface areas have attracted extensive attention and great interest from researchers in the field of separation science. Carbon dots (CDs), a new type of zero-dimensional fluorescent carbon nanomaterials, have been widely used in bioimaging, light-emitting diodes, sensing, catalysis, drug delivery, and other applications since they were first reported in 2004. These nanomaterials present several advantages over other types of separation materials, including a simple preparation process, low toxicity, easy functionalization, excellent biocompatibility, and photobleaching resistance. In addition, compared with traditional carbon nanomaterials such as graphene and carbon nanotubes, CDs have abundant surface functional groups, nanoscale sizes, and moderate adsorption properties. Hence, when CDs-based new materials are applied as a stationary phase for column chromatography, they can provide rich reaction sites and ensure the uniformity of the chromatographic packing process. The use of CDs can effectively avoid the peak-tailing phenomenon caused by the strong interaction of large *π*-conjugated systems with some analytes and improve the efficiency of the chromatographic column. As such, these nanomaterials show good application prospects in the field of chromatographic separation. In this review, the development history, classification, and synthesis strategies of CDs are briefly described. We then focus on the development of CDs-based chromatographic separation materials by systematically reviewing the recent advances in the use of CDs-based materials as a stationary phase for high-performance liquid chromatography (including hydrophilic interaction, reversed-phase, mixed-mode, and chiral chromatography), gas chromatography, and capillary electrochromatography, with special emphasis on the preparation methods and applications of various stationary phases. Finally, the development prospects of CDs and future research efforts on these materials are also analyzed and discussed. This review can provide guidance on the rational design and application of new CDs-based chromatographic separation materials.

碳点(carbon dots, CDs),作为一类具有显著荧光性能的零维准球形碳纳米材料,是继石墨烯和碳纳米管之后的21世纪碳材料家族中的新星^[1,2]^。CDs具有合成简单、稳定性好、成本低、粒径大小可调、易于功能化以及低毒等优点,被广泛应用于生物成像、抗菌、信息加密、药物递送、催化、光电器件和分析检测等领域^[3⇓⇓⇓⇓⇓-9]^。

## 1 CDs的发展历程

在Web of Science中输入关键词“Carbon dots”,通过文献出版量可将CDs的发展历程大致分为3个阶段:发现阶段(2004-2006)、发展初期(2006-2012)及快速发展阶段(2012至今),其中发现阶段是CDs被发现和定义的阶段(图1)。历史上,很多重大的科学发现都产生于偶然。2004年,Xu等^[10]^在电泳纯化分离单壁碳纳米管悬浮液的过程中偶然发现“荧光碳纳米颗粒”; 2006年,Sun等^[11]^用激光刻蚀策略制得荧光碳纳米颗粒,并将其正式定义为“碳量子点”。之后研究人员才对这一领域逐渐产生兴趣。使用“CDs”这一命名是为了准确地将它们与更广泛的碳纳米颗粒领域(如炭黑)区分开来^[12]^。从那时起,关于CDs性质的研究开始零星报道,但关于CDs的基础知识仍然扑朔迷离。

**图 1:**
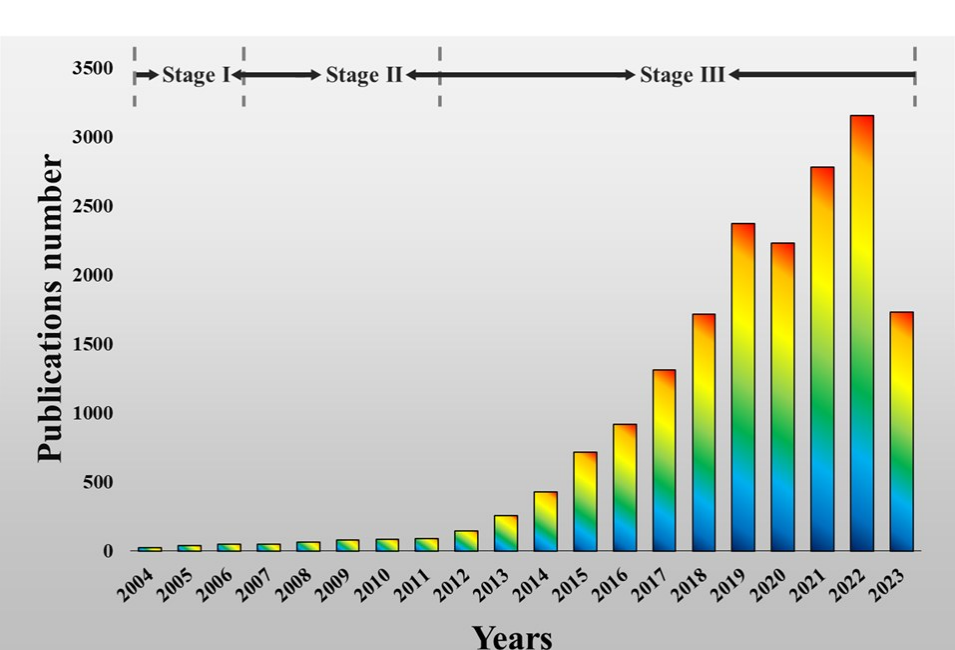
碳点的文献出版量

第二阶段,主要是对CDs的合成策略进行了大量探索,比如:电化学/化学氧化策略、软模板法、水热/溶剂热合成法、超声合成法等^[13⇓⇓⇓-17]^。更有趣的是,在这个阶段研究人员还发现改变实验参数(如引入杂原子、改变反应溶剂等)可以调控CDs的光电化学性质,从而激发了更多科研工作者对CDs构效关系的研究。也是在这个阶段,CDs在各个领域的应用研究得到了广泛关注。

第三阶段,从2012年开始,与CDs相关的出版物数量开始出现指数级增长,除了进一步拓展CDs的应用领域外,其他关于CDs的新合成策略以及规模化制备、商业化应用也被大家关注,以及长期困扰大家的CDs的荧光机制^[18⇓-20]^。目前报道的主要有以下几种荧光机制:量子限域效应、量子尺寸效应、表面状态/分子(荧光团)状态和交联增强发射效应^[6]^。

## 2 CDs的分类与合成策略

CDs通常是由纳米晶型/无定型的*sp*^2^-*sp*^3^杂化碳核和丰富的表面官能团(如羟基、羧基、氨基、醛基、巯基等)组成。根据碳核和附着状态的不同一般可将CDs分为五大类^[21]^:石墨烯量子点(graphene quantum dots, GQDs)、碳纳米点(carbon nanodots, CNDs)、碳量子点(carbon quantum dots, CQDs)、碳化聚合物点(carbonized Polymer dots, CPDs)和石墨氮化碳量子点(graphitic carbon nitride quantum dots, g-CNQDs)。GQDs通常包含单层或多层小片的石墨结构,来源于石墨烯或氧化石墨烯,具有明显的石墨晶格和各向异性(纵向尺寸约2.5 nm、横向尺寸约20 nm)^[22]^。CNDs不具备典型的晶格结构(多为无定型),碳化程度很高,表面官能团较少^[23]^。CQDs通常由非晶碳构成,碳化程度不及CNDs的碳化程度高,具有丰富的表面官能团和激发依赖性,量子尺寸效应突出,荧光发射波长可覆盖可见-近红外区^[24]^。CPDs是聚合物前体通过脱水聚合、交联和碳化得到的交联/碳化产物,通常为内部的碳核与外部的聚合物链(壳)通过自组装形成的“核-壳”结构^[25]^。g-CNQDs则是由氨基桥接的三-s-三嗪环组成,其晶格中存在周期性空位^[26]^。

CDs的合成策略主要包括“自上而下”法和“自下而上”法^[1]^。其中“自上而下”法主要采用大尺寸的碳材料前体(如石墨、金刚石等)通过一系列物理、化学或电化学操作获得,主要包括电弧放电、激光消蚀、电化学剥离和化学氧化法等,该策略的操作过程较为复杂,反应条件相对苛刻,发光量子产率低(通常需要进行表面化学修饰来改善荧光量子产率),但易于规模化制备;而“自下而上”法则是将有机单体和/或聚合物通过聚合、脱水碳化等方式获得,主要包括水热/溶剂热法、直接热解法、超声合成法、微波辐射法等(图2)。这种合成策略具有丰富且廉价的原料来源,且具有操作简便、产物量子产率高、反应条件温和等优势,在CDs的合成领域备受欢迎,但该策略副产物多,不适合制备具有精确结构的CDs。目前,CDs的合成主要还是采用试错法进行,非常耗时。为了克服这一不足,研究人员首先使用机器学习(machine learning)对CDs合成过程的重要参数(如温度、前驱体种类和浓度、反应溶剂、反应时间等)进行优化,再与“自上而下”或“自下而上”的合成方法结合,大大提高了合成效率^[7]^(见图3)。

**图 2:**
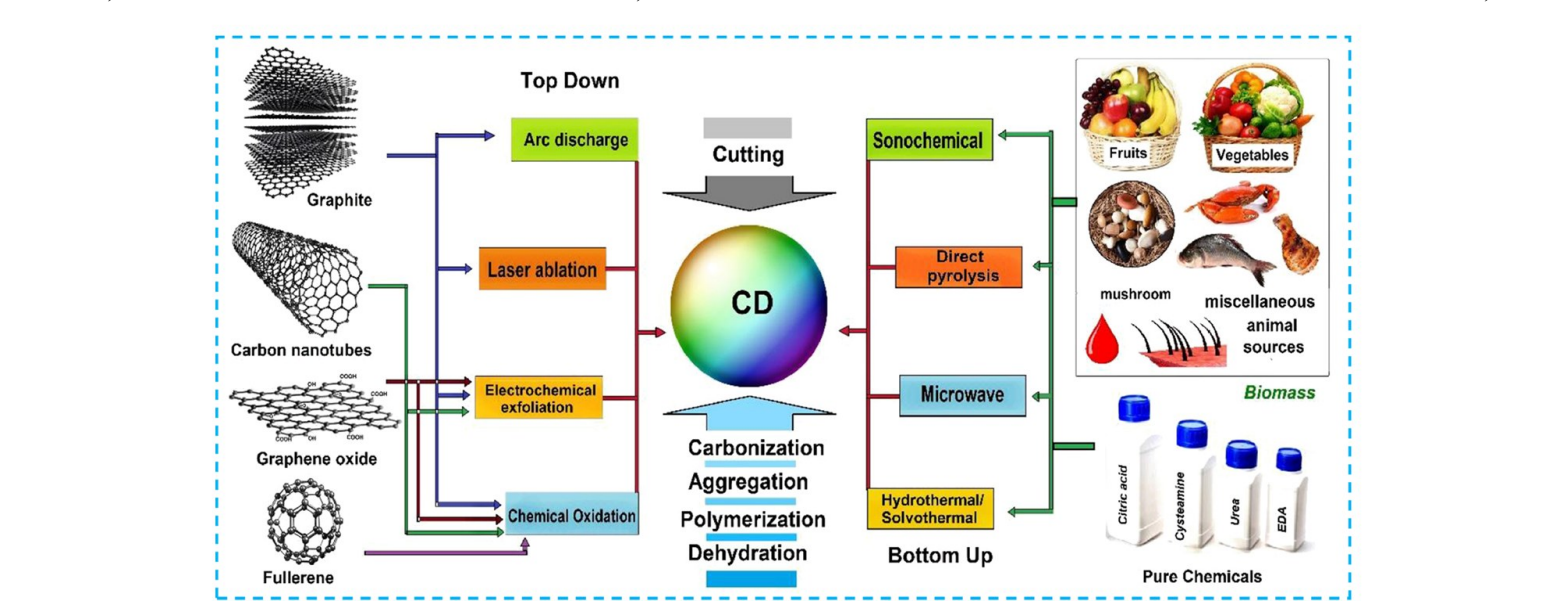
CDs的主要合成策略及形成机理^[1]^

**图 3:**
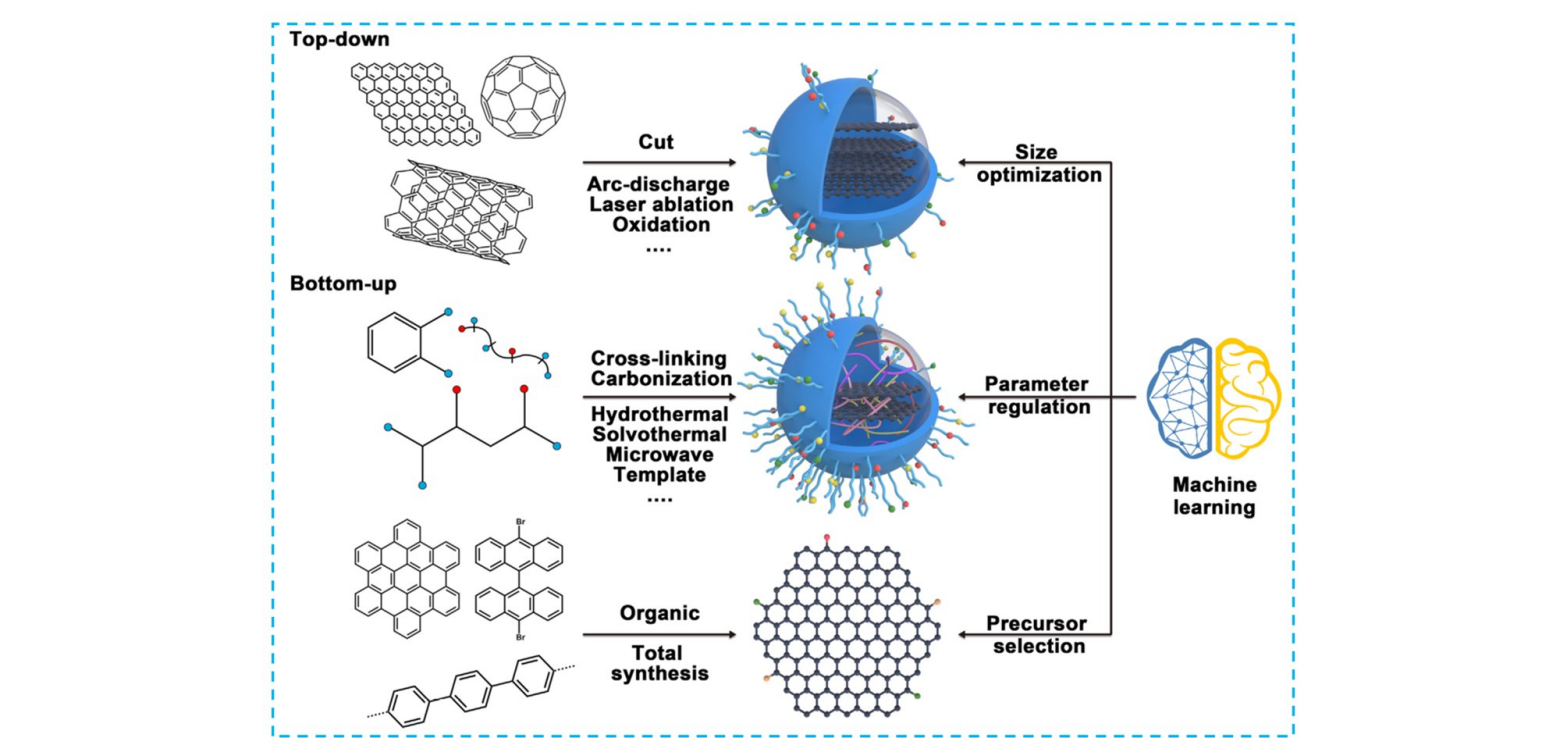
机器学习结合“自上而下”或“自下而上”合成法用于CDs的制备^[7]^

色谱分离是利用待分离组分在固定相和流动相构成体系中的分配系数差异达到分离的目的,具有分析速度快、分离效率高、样品用量少、灵敏度高、选择性好等优势,是分析化学领域最富活力、应用最为广泛的分离分析方法之一^[27]^。色谱分离的核心是色谱柱,色谱柱的灵魂是色谱分离材料,色谱分离材料的种类和性质直接决定色谱的分离模式和分离性能^[28]^。CDs作为一类新型的明星材料,在色谱分离材料的可控制备方面发挥着重要角色和应用潜力。本文重点介绍了CDs在液相色谱(LC)、气相色谱(GC)和毛细管电色谱(CEC)固定相方面的最新研究进展。在此基础上,展望了CDs在色谱分离领域的发展前景和努力方向,以期为基于碳点的色谱分离新材料的合理设计、精准调控及应用提供一些参考。

## 3 CDs在色谱固定相中的应用

CDs与传统的石墨烯、碳纳米管等碳纳米材料相比,具有丰富的表面官能团、较小的粒径和适中的吸附性能,因此在其应用到色谱固定相时,可提供丰富的反应位点,保证装柱过程色谱填料的均匀性,并有效避免大的*π*共轭体系对某些分析物的强相互作用而造成的峰拖尾现象,进而提高柱效,可在色谱分离领域展示出良好的应用前景^[29]^。

### 3.1 CDs在液相色谱固定相中的应用

高效液相色谱是一种广泛使用的分离分析技术。其中,亲水作用色谱(HILIC)于1990年由Alpert提出,适用于强极性或亲水性成分的分离,在食品、药物、环境科学等领域广泛使用。2012年,Li等^[30]^采用硝酸处理玉米秸秆燃烧后的灰烬,得到尺寸小于18 nm的碳纳米颗粒,并将其成功键合到多孔硅胶表面,用于HILIC和富水液相色谱(per aqueous liquid chromatography, PALC)模式下5种核苷、4种磺胺和实际样品红花注射液的高选择性分离。之后,本课题组围绕CDs开展了多种CDs(如:色氨酸和乌头酸衍生CDs^[31]^、咪唑离子液体衍生CDs^[32]^、对苯二胺衍生CDs^[33]^、葡萄糖衍生CDs^[34]^、氮掺杂葡萄糖衍生CDs^[35]^、硅烷化CDs^[36]^、聚乙烯亚胺衍生CDs^[37]^、四乙烯五胺衍生CDs^[38]^)键合硅胶亲水色谱固定相的研究工作。Yuan等^[34]^以葡萄糖为碳源,采用水热合成法制备葡萄糖衍生CDs,并以硅烷化试剂作为偶联剂,通过“nano-on-micro”策略,将其键合在硅胶表面,得到葡萄糖衍生CDs键合硅胶色谱固定相。结果表明,该固定相可在HILIC模式下实现氨基酸、糖类、人参皂苷、抗生素和碱基核苷等化合物的高选择性分离。实验还发现,葡萄糖衍生CDs键合硅胶色谱固定相较于葡萄糖分子直接键合硅胶色谱固定相有更好的分离性能,这可能是由于葡萄糖分子与硅胶的相互作用位点少于葡萄糖衍生CDs和硅胶的相互作用位点所致。另外,该固定相可实现枸杞果实提取液中果糖和葡萄糖的定性和定量分析。杂原子掺杂策略可以进一步改变材料的化学组成和结构特性^[39]^,为了验证上述思想,本课题组^[35]^在葡萄糖衍生CDs的制备过程中加入了天冬氨酸,成功制备出氮掺杂葡萄糖衍生CDs(Glc-NCDs),随后以3-异氰酸丙基乙氧基硅烷作为硅胶表面的硅烷偶联剂,将氮掺杂葡萄糖衍生CDs键合至硅胶表面,得到氮掺杂葡萄糖衍生CDs键合硅胶色谱固定相(Sil-Glc-NCDs,图4),该固定相在分离氨基酸、糖类、人参皂苷和抗生素时,表现出比葡萄糖衍生CDs键合硅胶色谱固定相更优异的分离选择性,且可用于罗红霉素胶囊中罗红霉素的定量测定。

**图 4:**
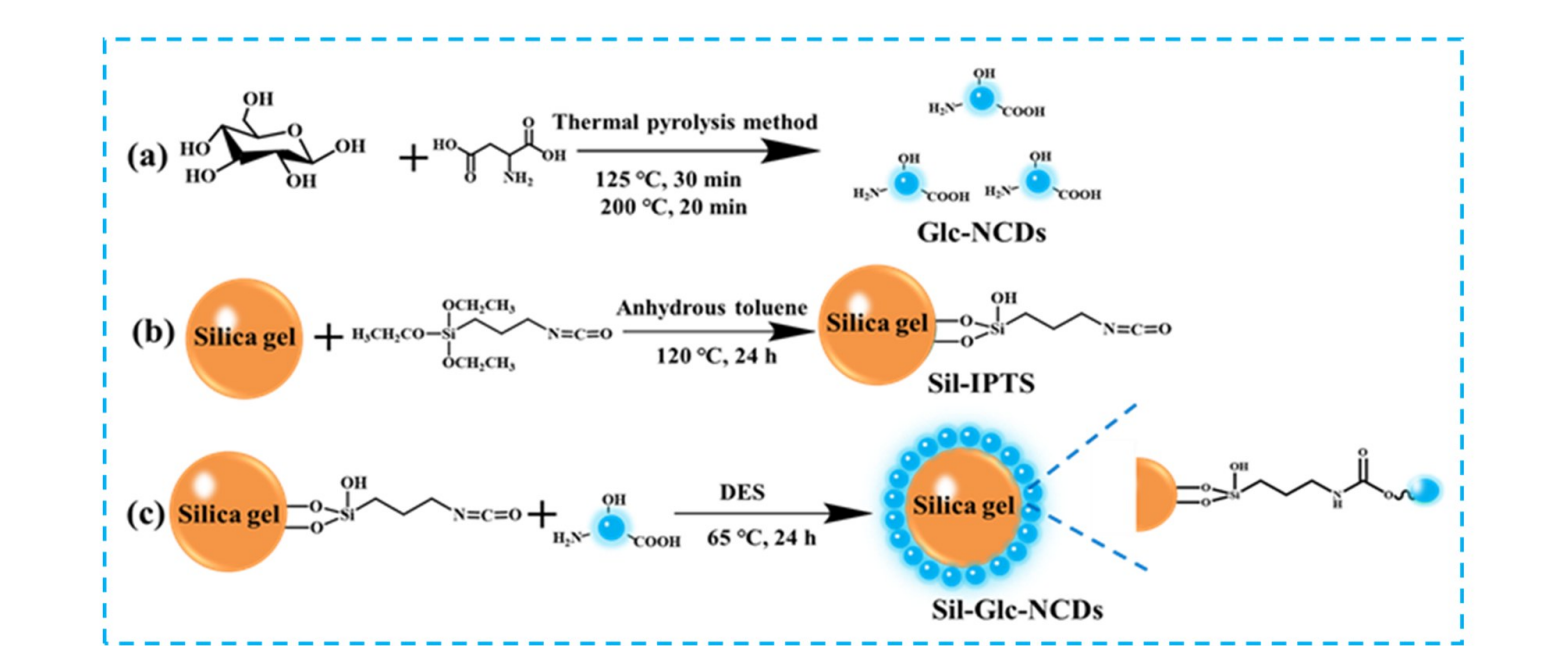
Sil-Glc-NCDs固定相的制备示意图^[35]^

为了简化固定相的制备流程,研究人员选用柠檬酸和*N*-(*β*-氨乙基)-*γ*-氨丙基甲基二甲氧基硅烷(*N*-(*β*-aminoethyl)-*γ*-aminopropyl-methyldimethoxysilane, AEAPMS)热解反应合成出带有硅烷基团的氮、硅共掺杂碳点,直接与硅胶键合,制得氮、硅共掺杂CDs键合硅胶色谱固定相(Sil-CDs)^[36]^。该固定相的制备过程不需要额外引入硅烷化试剂,操作更为简便快速,可在HILIC模式下实现9种磺胺、10种碱基核苷、7种黄酮和7种氨基酸的高选择性分离。通过对色谱保留机理进行考察,发现该固定相的色谱保留能力主要依靠静电相互作用和亲水分配作用实现,而这两种相互作用均源于碳点的表面修饰剂AEAPMS的化学性质。另外,相较于AEAPMS直接键合的硅胶色谱固定相(Sil-AEAPMS), Sil-CDs表现出更优异的色谱分离性能,其色谱性能的提升再次验证了CDs的形成使固定相表面存在更多的相互作用位点。Cai等^[38]^以四乙烯五胺作为制备CDs的前驱体和反应溶剂,在高温下加入柠檬酸裂解反应得到表面带有前驱体基团的CDs和前驱体的混合物。随后,将上述混合物直接硅烷化处理后键合到硅胶表面,制得带有前驱体基团的CDs和前驱体共键合硅胶亲水色谱固定相,用于碱基核苷、氨基酸和人参皂苷等亲水性物质的选择性分离。研究表明,该方法制备的共键合硅胶色谱固定相具有比其前体键合硅胶固定相更丰富的表面官能团,可为亲水色谱模式下样品的分离提供更强的亲水分配作用和吸附作用。

此外,Luo等^[40]^还将生物大分子牛血清白蛋白修饰到GQDs键合的硅胶表面,成功制备了双亲水物质牛血清白蛋白修饰的GQDs键合硅胶色谱固定相(BSA@GQDs@SiO_2_,图5),结果发现其可在HILIC模式下,实现核苷碱基、酸类化合物、苯酚、奎诺酮、维生素以及生物碱的分离,且其分离性能优于商品化的氨基柱。

**图 5:**
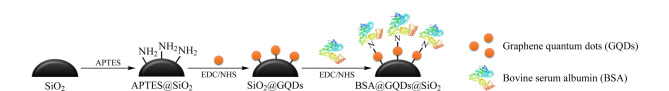
BSA@GQDs@SiO_2_固定相的制备示意图^[40]^

分子印迹聚合物(MIPs)具有选择性高、稳定性好、使用寿命长、抗恶劣环境能力强等优点,广泛应用于色谱分离、固相萃取、催化、传感等领域^[41⇓-43]^。Chai等^[44]^利用MIPs的高选择性以及CDs出色的物理化学性质,通过悬浮的方法成功制备了MIP-CDs/SiO_2_色谱固定相,用于核苷、磺胺、苯甲酸和抗生素的分离,其分离性能显著优于MIP/SiO_2_、CDs/SiO_2_色谱柱以及常用商品柱(图6)。

**图 6:**
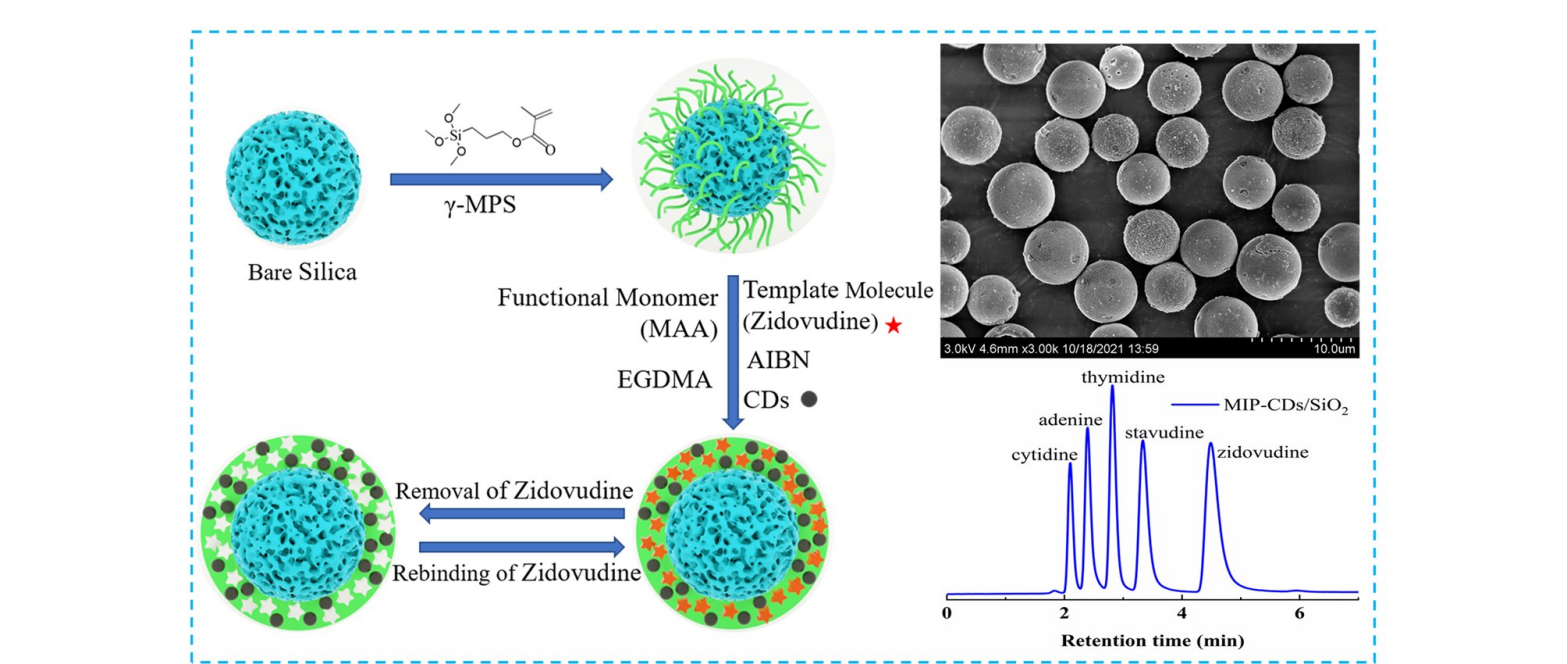
MIP-CDs/SiO_2_固定相的合成、电镜表征及色谱性能^[44]^

此外,本课题组还利用CDs设计性强的优势,成功制得系列CDs(如十八烷基胺掺杂葡萄糖衍生CDs^[45]^、疏水长链咪唑离子液体衍生CDs^[46]^、低共熔溶剂衍生CDs^[47]^)键合硅胶反相液相色谱填料。Chen等^[45]^在葡萄糖衍生CDs的制备过程中引入疏水长链十八烷基胺作为CDs的表面修饰剂,得到十八烷基胺掺杂葡萄糖衍生CDs (Glc-OCDs),与3-(2,3-环氧丙氧)丙基三甲氧基硅烷键合最终制得CDs键合硅胶色谱填料(Sil-Glc-OCDs,图7),该固定相可在反相色谱模式(RPLC)下实现7种多环芳烃、8种烷基苯的良好分离,特别是对叔丁基苯、仲丁基苯、异丁基苯和正丁基苯4种同分异构体表现出优异的分离选择性。该固定相还可用于低共熔溶剂处理的黄芪药材中6种黄酮类化合物的定性和定量检测。

**图 7:**
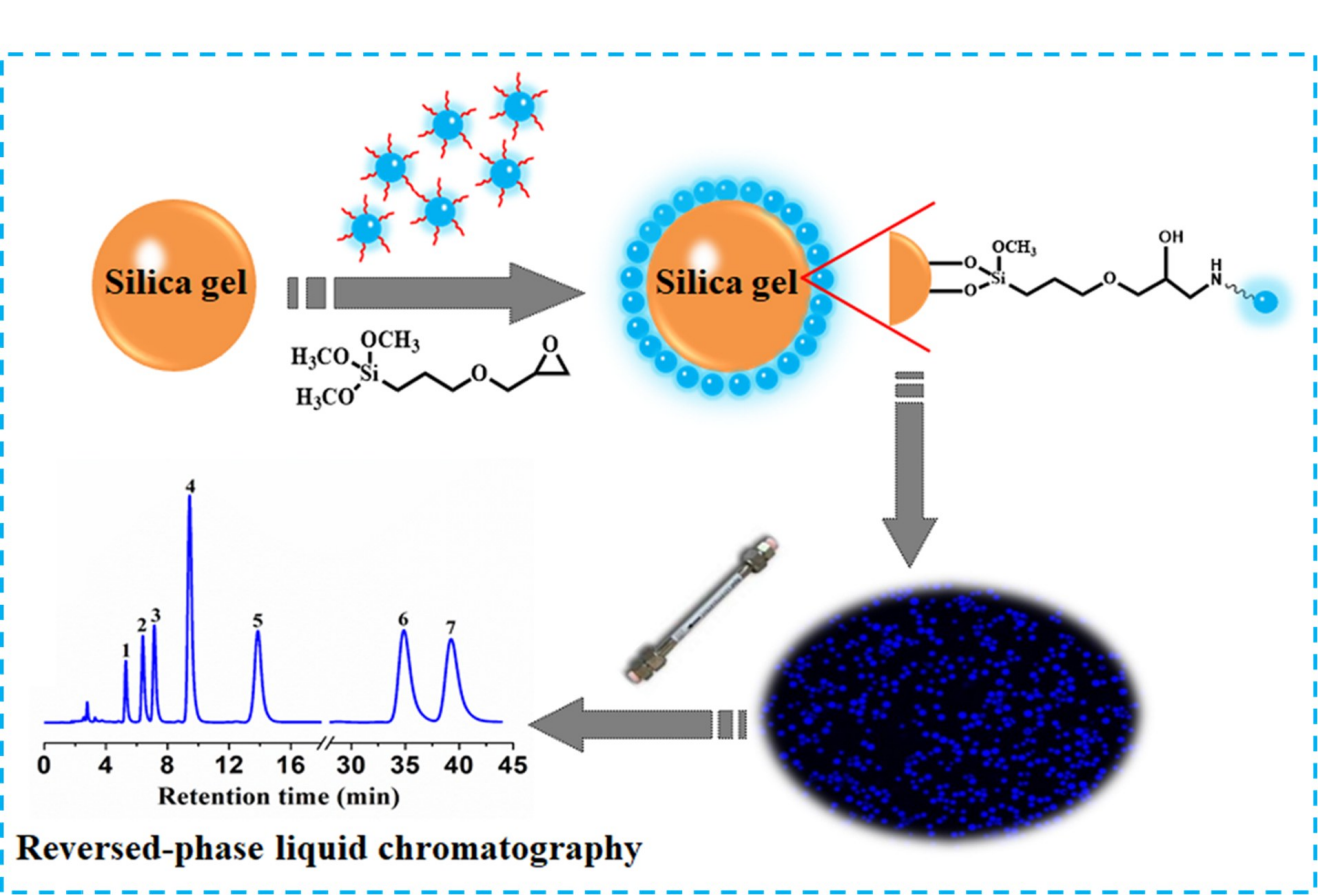
Sil-Glc-OCDs固定相的制备及对7种多环芳烃的分离^[45]^

离子液体(ILs)是由有机阳离子和无机或有机阴离子构成的在室温或低温(< 100 ℃)条件下呈液态的有机盐,又称为室温离子液体,具有良好的溶解性、稳定性和结构可设计性,可作为疏水碳点的理想原料^[48]^。Jiang等^[46]^以1-乙烯基-3-十八烷基咪唑溴盐([C_18_VIm]Br)离子液体为碳源,成功制备了ILs衍生CDs键合硅胶反相色谱固定相(Sil-ImC_18_CDs)、ILs键合硅胶反相色谱固定相(Sil-ImC_18_)、ILs衍生CDs和ILs共键合硅胶反相色谱固定相(Sil-ImC_18_/CDs)。元素分析结果表明,Sil-ImC_18_/CDs固定相相较于Sil-ImC_18_、Sil-ImC_18_CDs有更高的键合密度。同时,Sil-ImC_18_/CDs固定相表现出比Sil-ImC_18_和Sil-ImC_18_CDs固定相更优异的分离性能,可成功实现4种4个环/3个环的多环芳烃以及丁苯异构体的高选择性分离,这可能是由于咪唑ILs提供的*π*-*π*堆积作用、十八烷基链的强疏水相互作用以及ImC_18_CDs和[C_18_VIm]Br间的协同效应所致。另外,Sil-ImC_18_/CDs固定相表现出良好的柱稳定性,可成功用于甲醇处理的黄芪提取液中4种黄酮类化合物的定性和定量分析。该工作不仅为CDs键合硅胶反相色谱固定相的制备提供了一种新思路,同时也对键合量的提升具有重要指导意义。

2003年,英国莱斯特大学Abbott教授提出了一种新型离子液体——低共熔溶剂(deep eutectic solvents, DESs), DESs主要是由一定物质的量比的氢键受体和氢键供体通过氢键作用结合而成的一种低共熔混合物,具有低毒、可生物降解、制备简单、价格低廉、结构可设计等优势,被认为是新型绿色溶剂,在诸多领域备受广大科研工作者的青睐^[49⇓-51]^。2022年,Fu等^[47]^以氯化胆碱为氢键受体与乳酸为氢键供体组成的DESs为CDs的前驱体,成功制备DESs衍生CDs键合硅胶色谱固定相。采用Tanaka和Engelhardt标准测试物考察该固定相的保留行为,发现其表现出良好的疏水作用、*π*-*π*作用和氢键作用等,可在反相模式下实现11种多环芳烃、12种芳香胺、5种烷基苯、7种酚类和6种黄酮类化合物的高选择性分离。更有意思的是,该固定相可构建绿色色谱,在纯水作流动相的情况下,实现结构类似物氢化可的松和泼尼松龙的基线分离。

混合模式色谱固定相是一种能够发生多种相互作用模式的色谱固定相,相较于单一模式的色谱固定相,在分离极性、非极性化合物等复杂样品方面具有很好的灵活性,在色谱分析中发挥重要作用。Fu等^[52]^以氯化胆碱与乳酸组成的DESs和磷酸为原料制备出磷掺杂DESs衍生CDs(P-DESCDs),采用“nano-on-micro”策略将P-DESCDs键合到硅胶表面形成磷掺杂DESs衍生CDs键合硅胶固定相(Sil-P-DESCDs),通过对Sil-P-DESCDs固定相的保留行为进行考察,发现其适用于RPLC和HILIC的混合模式。可在RPLC模式下,实现6种烷基苯、12种多环芳烃、5种磺胺、8种芳香胺、6种苯酚以及6种黄酮类化合物的高效分离,其分离性能可与未掺杂磷的DESs衍生CDs键合硅胶色谱固定相媲美;在HILIC下,可实现9种碱基核苷和7种生物碱的分离(图8)。该固定相具有良好的柱稳定性,可用于药品黄芪颗粒中毛蕊异黄酮葡萄糖苷的分析检测。此外,Wu等^[53]^以柠檬酸和1,8-二氨基辛烷为原料合成两亲性CDs,并键合至硅胶表面作为RPLC和HILIC混合色谱固定相。通过线性溶剂化方程考察了该固定相的保留机理,该固定相可用于多环芳烃、烷基苯、碱基核苷、氨基酸、*β*-肾上腺素受体、磺胺、抗生素和生物碱等多种成分的高效分离。当然,柠檬酸和十八烷基胺也可作为CDs的碳源,构建RPLC和HILIC混合色谱固定相,用于RPLC模式下多环芳烃和烷基苯的分离,HILIC模式下磺胺、核苷等化合物的高选择性分离,以及实际样品牛奶中氯霉素的检测^[54]^。

**图 8:**
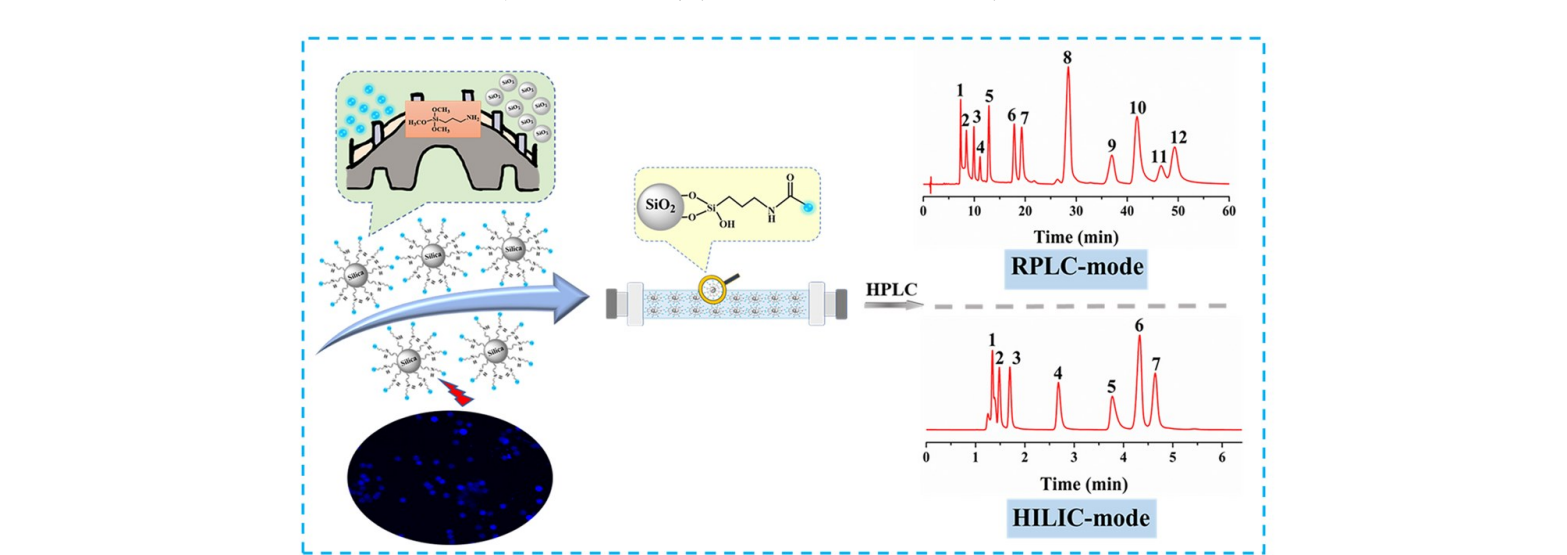
Sil-P-DESCDs固定相的制备流程及色谱性能^[52]^

GQDs作为CDs的子集,以其为硅胶基质色谱固定相表面键合相时可与分析物之间形成疏水、氢键、亲水、*π-π*堆积等多重相互作用,可在RPLC模式下实现多环芳烃、苯胺和苯酚的高选择性分离,HILIC模式下实现生物碱、核苷和碱基的分离^[55]^。由于ILs对酸、碱化合物均具有良好的分离性能,为了提升GQDs键合硅胶色谱固定相对酸性化合物的分离性能,Wu等^[56]^发展了功能分子1-氨基乙基-3-甲基咪唑溴ILs修饰的GQDs键合硅胶色谱固定相,成功构建了离子交换色谱(IEC)、HILIC和RPLC多种色谱分离模式。此外,十八烷基胺也被修饰到GQDs键合硅胶表面,用于HILIC和RPLC混合色谱固定相的构建,实现苯胺、苯酚、多环芳烃、烷基苯、生物碱、核苷碱基的高效分离^[57]^。

对映体分离一直是分离科学中的一个难题,其核心仍然是高选择性的手性固定相的设计合成^[58]^。Wu等^[59]^发展了纤维素功能化的GQDs键合硅胶色谱固定相以及*β*-CD功能化的GQDs键合硅胶色谱固定相,并详细考察了两种固定相的手性拆分性能。结果发现当手性选择剂与对映体作用时,GQDs的引入可提供额外的相互作用,进而显著提升固定相的对映体拆分性能,这就为新型手性色谱分离材料的制备提供了新的思路。

### 3.2 CDs在气相色谱固定相中的应用

GC具有分析速度快、灵敏度高、应用范围广、选择性好、试剂消耗少等优点^[60]^。由于CDs粒径较小,将其作为气相色谱固定相时,有利于改善传质速度。Zhang等^[61]^以氨丙基二乙氧基甲基硅烷为偶联剂,将GQDs键合在毛细管内壁成功制得中等极性的GQDs键合毛细管气相色谱柱,通过GQDs与分析物之间的范德华力与*π*-*π*作用,实现二甲苯、丙苯、二氯苯、烷烃、苯乙烯异构体、乙苯等化合物的高选择性分离,并且该过程无需程序升温。在低温环境下GQDs键合毛细管气相色谱柱相较于商品化的HP-5和HP-innowax毛细管柱具有更快的分析速度。

### 3.3 CDs在毛细管电色谱固定相中的应用

CEC是CE和HPLC相结合的一种分离分析技术,兼具高柱效、高分辨率、高选择性和高峰容量的特点,同时具有色谱和电泳的双重分离机理。其中,毛细管柱是CEC分离的核心部件,依据色谱柱内壁固定相的不同形式可将毛细管柱分为开管毛细管柱、填充毛细管柱和整体毛细管柱。Fu等^[62]^在3-氨丙基三乙氧基硅烷(3-aminopropyltriethoxysilane, APTES)和1,3,5-三(4-氨基苯基)苯(1,3,5-tris(4-aminophenyl)-benzene, TAPB)处理的毛细管内壁加入戊二醛为原料合成的表面富含醛基的CDs以及表面富含氨基的TAPB,通过原位席夫碱反应成功制备出CDs-共价有机纳米材料(CDs-based covalent organic nanomaterial, CON CDs-TAPB)改性的开管毛细管柱(图9),由于CON CDs-TAPB键合相在毛细管内壁可提供丰富的作用位点,故对烷基苯、氯苯、对羟基苯甲酸酯和酚类化合物表现出优异的分离性能(甲基苯的最大理论塔板数高达1.6×10^5^ plates/m)。另外,作者也对柱子的稳定性和重现性进行了考察,结果令人满意,这为CDs-共价有机纳米材料在毛细管电色谱中的应用提供了重要参考。

**图 9:**
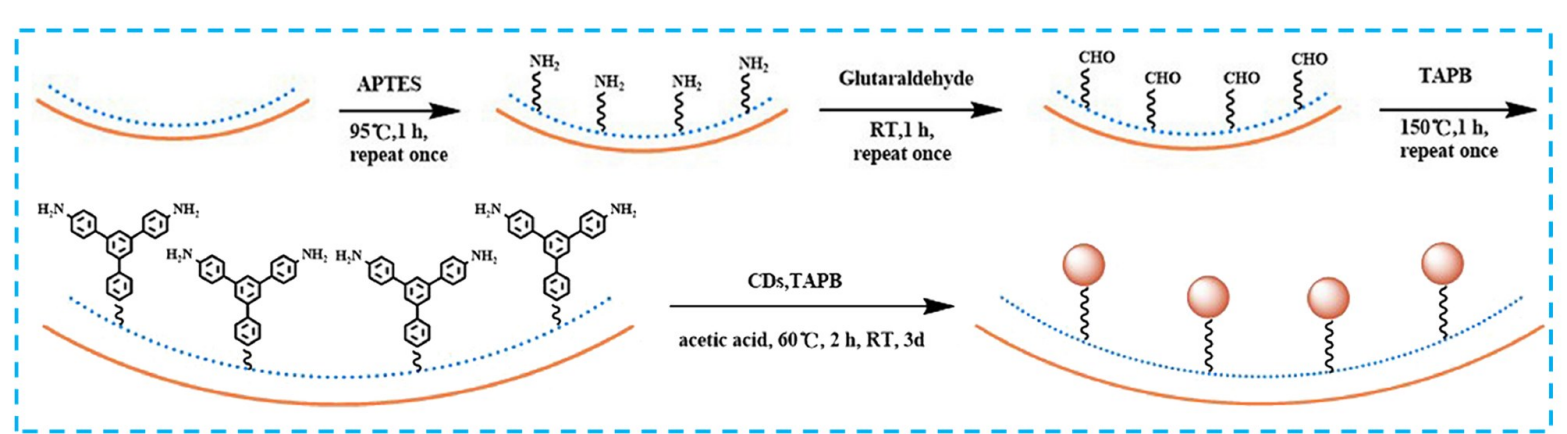
CON CDs-TAPB固定相的制备流程^[62]^

## 4 结论与展望

本文综述了CDs在色谱分离材料研究中的最新研究进展,主要涉及基于CDs的液相色谱固定相、气相色谱固定相和毛细管电色谱固定相的制备及应用。可以看到,由于CDs具有独特的物理化学性质,其在液相色谱固定相的研究中得到了广泛应用,对色谱分离性能的提升起到了显著作用,但其在气相色谱固定相和毛细管电色谱固定相中应用甚少,目前还处于起步阶段,这可能是由于碳点的合成产率普遍较低、纯化过程较为耗时等,未来仍有大量的研究工作值得探索。比如:进一步提升CDs的合成产率,简化基于碳点的色谱分离材料的制备和纯化流程,结合机器学习更有针对性地设计构筑新颖结构的CDs或CDs与其他功能材料的复合材料,拓宽CDs在分离材料领域的应用范围,解析CDs的结构对色谱分离性能的影响和作用机制等,以期进一步挖掘CDs的潜在应用价值。

## 作者团队简介

手性分离与微纳分析课题组(506组)隶属于中国科学院兰州化学物理研究所。自2012年以来, 课题组致力于离子液体、碳纳米材料、有机框架材料等新材料在复杂样品中结构类似物的分离分析(特别是手性分离、药物分离和稀土分离)方面的应用基础研究工作, 承担了多项国家级科研项目, 并与国内外相关领域学者建立了良好的合作关系。

课题组网站: http://www.licp.cas.cn/qhdz/

### 人才队伍

**课题组组长:** 邱洪灯研究员

**职工及学生:** 研究员1人, 副研究员1人, 助理研究员4人, 特别研究助理2人, 博士后及研究生20余人

**团队精神:** 胆大心细做学问, 奋发图强有色谱

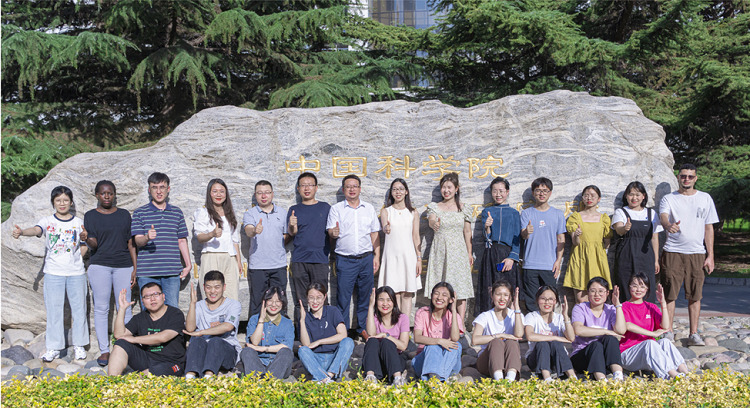



### 科研项目及成果

**科研项目:** 国家重点研发计划项目,国家自然科学基金(优秀青年基金,面上项目,青年基金),中国科学院西部之光交叉团队项目,甘肃省自然科学基金等

**科研成果:** 在*Adv. Funct. Mater.*, *Small*, *Anal. Chem.*, *J. Chromatogr. A*等期刊发表SCI论文200余篇, 引用7000余次, 申请国家发明专利50余项, 论著5章

**获奖情况:** 中国分析测试协会科学技术奖(CAIA奖)一等奖,甘肃省自然科学奖二等奖,第六届“离子液体与绿色过程”青年创新奖与新秀奖,CCL优秀青年学者,兰州化物所青年创新奖特别奖等

### 研究领域

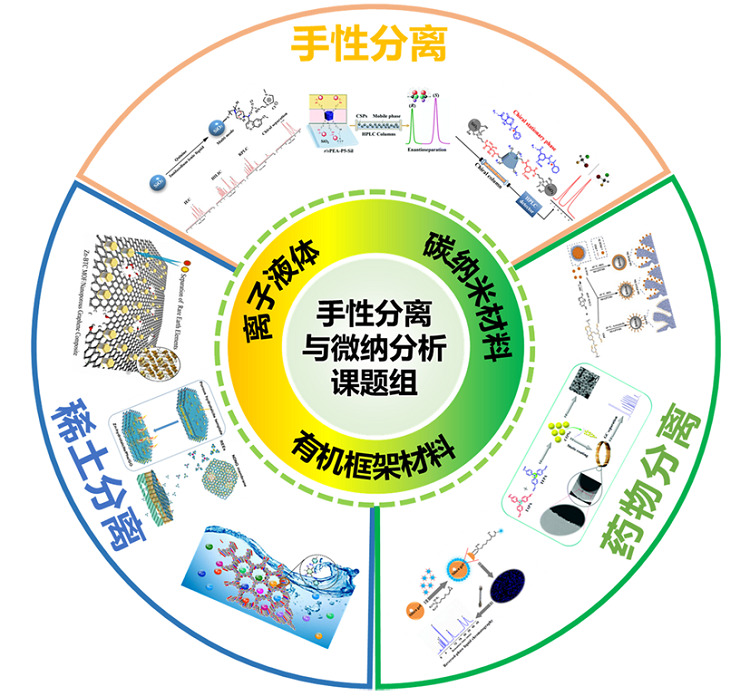



### 仪器设备

气相色谱-质谱仪,液相色谱-三重四极杆质谱联用仪,全自动液相色谱-微型质谱联用系统,全自动二维液相色谱系统,全功能稳态瞬态荧光光谱仪,紫外-可见-近红外分光光度计,各种高效液相色谱仪等,共20余台/套


